# Current Development, Obstacle and Futural Direction of Induced Pluripotent Stem Cell and Mesenchymal Stem Cell Treatment in Degenerative Retinal Disease

**DOI:** 10.3390/ijms23052529

**Published:** 2022-02-25

**Authors:** Ming-Cheng Chiang, Edward Chern

**Affiliations:** niChe Lab for Stem Cell and Regenerative Medicine, Department of Biochemical Science and Technology, National Taiwan University, Taipei 10617, Taiwan; csmucell1223@gmail.com

**Keywords:** induced pluripotent stem cells, mesenchymal stem cell, retinal disease, review article

## Abstract

Degenerative retinal disease is one of the major causes of vision loss around the world. The past several decades have witnessed emerging development of stem cell treatment for retinal disease. Nevertheless, sourcing stem cells remains controversial due to ethical concerns and their rarity. Furthermore, induced pluripotent stem cells (iPSCs) and mesenchymal stem cells (MSCs) are both isolated from patients’ mature tissues; thus, issues such as avoiding moral controversy and adverse events related to immunosuppression and obtaining a large number of cells have opened a new era in regenerative medicine. This review focuses on the current application and development, clinical trials, and latest research of stem cell therapy, as well as its limitations and future directions.

## 1. Introduction

The retina measures 0.5 mm thick and is composed of several radial portions. Retinal ganglion cells (RGCs) are located at the inner layer of the retina, while photosensors (rods and cones) are in the outermost layer close to the sclera [1]. The retinal pigment epithelium (RPE) is a monolayer of hexagonal cells that support photosensor survival, form tight junctions between the subretinal space and choriocapillaris as a barrier and maintain an ionic gradient, and facilitate phototransduction. The RPE plays a crucial role in photosensor survival by transporting nutrients to the photosensors, extracting waste, and renewing photosensors with phagocytosis of shedded outer segments. Through its role in ionic passage regulation and its asymmetric distribution of specialized transport proteins, the RPE controls the pH level, fluid transportation, and hyperpolarization/polarization of cell membranes to create a suitable microenvironment nourishing the photosensors [2,3,4]. In summary, the RPE is an irreplaceable part of vision formation, and severe visual impairment may occur if the RPE is damaged. Whether the number or density of RPE cells decreases with age is still controversial. Panda-Jonas et al. indicated that the RPE cell density decreases by 0.3% per year, while another study suggested that the density of RPE cells in the macula remains unchanged as peripheral RPE cells may migrate to the macula and compensate for macular cellular death [5,6]. However, clinical observations have revealed poor regenerative ability of the RPE [7]. Thus, degeneration or death of RPE cells is difficult to repair and regenerate. As a result, the RPE has received increasing attention in regenerative medicine.

Degenerative disease retinal diseases including age-related macular degeneration (AMD), diabetic retinopathy (DR), retinitis pigmentosa (RP), and Stargardt’s disease (Stargardt’s macular dystrophy, STGD) are caused by degeneration and death of photosensors or their underlying supportive layer, the RPE [8]. Anti-vascular endothelial growth factor (VEGF) therapies including intravitreal ranibizumab or bevacizumab and photodynamic therapy have been used to deal with neovascularization in DR and AMD [9,10,11,12]. Unfortunately, the abovementioned therapies have been proven to be effective but require repeated doses, which are distressing to patients; furthermore, invasive drug delivery exposes them to ocular complications ranging from subconjunctival hemorrhage to endophthalmitis [13,14]. With regard to inherited diseases, only supportive management to slow disease progression has been widely recognized, and novel treatments such as gene therapy or visual cycle modulators are still under survey [15,16]. The emergence of stem cell therapy has aimed to replace degenerative tissue that is beyond management and rarely recoverable with different stem cell sources from embryonic stem cells (ESCs) to mesenchymal stem cells (MSCs). As research on novel technology for stem cell therapy has been published, more valuable and interesting features of stem cells have raised interest in regenerative medicine.

Yamanaka et al. successfully reprogrammed somatic cells to a pluripotent state; these cells are known as induced pluripotent stem cells (iPSCs). iPSCs have growing potential and share the same properties of embryonic stem cells; furthermore, they are free from ethical issues [17]. iPSCs can be induced into organoids to replace injured tissues. In addition, iPSCs contribute to disease model establishment, drug screening, and personalized treatment planning, breaking new ground in stem cell therapy. Although they possess several advantages, more studies focusing on overgrowth and tumorgenicity of iPSCs are necessary. MSCs are another source of stem cells that are multipotent stromal cells isolated from bone marrow, adipose tissue, dental pulp, and amnionic fluid; they have the capacity to differentiate into specific cell types [18]. In addition to the function of replacing injured tissue with MSC-derived organoid, the cytokines, exosomes, and other secretomes of MSCs have been studied in various emerging applications and have advanced stem cell therapy to a new level. To date, a growing number of molecular and cellular biology findings regarding stem cells have been reported and applied to clinical treatment.

## 2. Induced Pluripotent Stem Cell

### 2.1. Current Application and Development

Takahashi et al. have reprogrammed adult fibroblasts into iPSCs through transduction of specific transcription factors. Twenty-four gene candidates were selected to induce pluripotency in somatic cells, and an assay system detecting the pluripotent state was created. By withdrawing a single gene in a cultural pool, they found that a lack of 10 genes would lead to no colony formation. Finally, four factors, namely, Oct3/4, Sox2, c-Myc, and Klf4, were found to produce a number of pluripotent colonies similar to those created with the pool of 10 genes, indicating that these four factors played key roles in iPSC generation [17]. With this outstanding achievement published, recent years have seen burgeoning applications of iPSCs to ophthalmic disease. Several characteristics of the human eye make it an ideal target for stem cell therapy: it is immunoprivileged as it can tolerate transplants, it can be monitored noninvasively, and the transplanted stem cells can be restricted in a relatively close environment [19,20]. In current studies, human iPSCs can be induced into retinal cells including the RPE, RGCs, and photosensors [21,22,23], providing novel therapy for AMD, STGD, RP, and myopia-induced macular degeneration, which are caused by gradual labefaction of photosensors and their underlying supporting tissue, the RPE. iPSC-derived RPE has been shown to be a potential candidate for replacement therapy, as it is able to transport ions, maintain a membrane potential, and secrete vascular endothelial growth factors similar to the native RPE [24].

Regarding clinical applications, Carr et al. transplanted iPSC-derived RPE as a cell suspension form into Royal College of Surgeons (RCS) dystrophic rats. Rats receiving iPSC-derived RPE transplantation showed better outcomes in visual acuity testing than those in the control group. They also confirmed that iPSC-derived RPE is functional and able to perform phagocytosis and fluid transport. Although the transplanted group lost their grafted cells 13 weeks later due to leukocyte infiltration, it was still an exciting outcome [25]. Assawachananont et al. transplanted iPSC-derived organoids in another form called a “retinal sheet”; the outcome showed formation of inner and outer segments of photosensor cells and a graft–host synaptic connection [26].

A human clinical trial was performed by Mandai et al. in which an autologous iPSC-derived RPE sheet without an artificial scaffold was transplanted in patients with AMD. Compared with the findings of previous study in which human ESC-derived RPE was transplanted into a patient with AMD but immunosuppressive-associated adverse events including decreased renal function and diarrhea occurred [27], patients receiving autologous iPSC-derived RPE transplantation showed no signs of rejection without immunosuppression. One year later, the patient had neither improvement nor decline in her best-corrected visual acuity, but an increased score on the Visual Function Questionnaire (VFQ-25) was noted [28]. Autologous iPSC-derived products have been shown to have no rejection response in monkey models, and iPSC-derived RPE cell sheets have morphological and physiological similarities to native RPE and improved traceability after transplantation compared with cell suspensions. Generating an RPE sheet without artificial scaffolding can reduce inflammation after surgery [29]. Since stable intraocular conditions have only been obtained in a single patient undergoing iPSC-derived RPE transplantation, more studies and trials are needed.

iPSC therapy can help us build disease models. Traditional disease models have been established in animals, which conceals interspecies variation and causes ethical controversy. iPSCs can differentiate into disease-relevant cell types carrying genomic defects, epigenetic markers, and pathological features. Owing to the novel “disease on the chip” model, drugs can be screened and personalized on the basis of sex, race, and mutation site patient by patient [30,31,32]. Moreover, it appeared to be a tool for genetic diagnosis of inherited retinal diseases [33]. The benefits of rapamycin, PP242, AICAR, NQDI-1, and salubrinal in photosensor cell survival have been discovered through an iPSC-derived RPE model from patients carrying a *rhodopsin* mutation [34]. Singh et al. built an iPSC-derived model of Best vitelliform macular dystrophy, known as abnormal accumulation of photosensor waste and bilateral macular degeneration. They found delayed rhodopsin degradation and impaired fluid transport, which led to autofluorescent material of breakdown products and oxidative stress accumulation as key factors of disease development [35].

**Figure 1:**
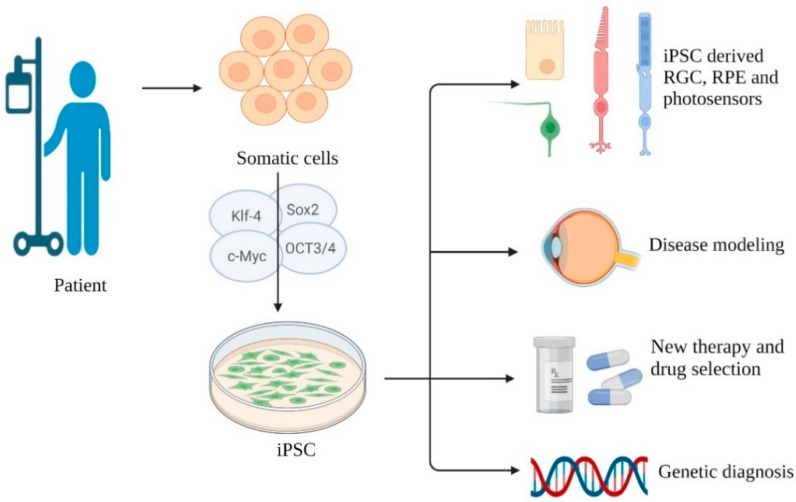
Somatic cells are isolated from the patient and reprogrammed with four transcription factors—Oct3/4, Sox2, c-Myc, and Klf4. iPSC, with pluripotent stemness, could be induced into RSC, RPE, and photosensor cells for replacement therapy. Disease model could be established for pathogenesis study. Research of drug selection, new therapy trials, and genetic diagnosis can be practiced on a patient-specific disease model derived from iPSC.

Above is a brief summary of iPSC development and applications in degenerative retinal disease (Figure 1). Next, we will discuss current limitations and novel ideas.

### 2.2. Safety and Tumorgenicity

Tumorigenicity is a major concern of iPSCs, including teratoma formation or overgrowth of the graft tissue. Several attempts have been made to remove potentially tumorigenic cells during and after the iPSC cell differentiation. Ogura et al. found that iPSCs pretreated with g-secretase inhibitor (GSI) developed into mature neuronal grafts, while control group samples showed overgrowth [36]. A highly sensitive way to locate residual undifferentiated cells employs a flow cytometry assay with anti-TRA-1-60 antibody and qRT-PCR with a specific probe, which has been published for tumorigenicity screening [37]. Past studies have suggested that a DNA methylation profile should be taken as a tumorigenicity evaluation criterion for iPSCs [38]. However, there are different opinions, such as that of Kanemura et al., who transplanted iPSC-derived RPE into immunodeficient mouse strains subretinally and then followed tumorigenicity for 36 weeks. By recording the minimal dose of transplanted cells that generated tumors in 50% of animals and considering the duration monitored, incidence of tumors, and number of rodents, they claimed that the tumorigenic potential of iPSC-derived RPE could be negligible [39]. Nevertheless, rodent models may not completely represent humans; furthermore, it is now more widely recognized that a detailed survey should be performed before transplantation for safety. Although it costs time and money to rule out all possibility of tumorgenicity, no immediate solution is available, and a more economic testing tool is needed.

### 2.3. Somatic Memory of Donor Site and the Role of Epigenetics

As mentioned before, iPSCs are reprogrammed from differentiated mature tissue. Some studies have demonstrated that transcription epigenetic memory from somatic cells is retained in iPSCs and that this memory may lead to variable differentiation efficiency [40,41,42]. Kim et al. found a relationship between the poor hematopoietic-forming potential of iPSCs derived from neural progenitor cells and epigenetic variations. iPSCs with better blood-forming ability show higher gene body methylation of *Wnt3* than those with poor hematopoietic potential, indicating that their low blood-producing ability may be caused by the persistent presence of suppressing epigenetic markers for blood formation or decreased expression of enhancing markers [43]. There is still a lack of consensus about which tissue should be harvested and reprogrammed. More studies are needed to explore the molecular mechanisms and effect of epigenetic memory as well as to establish a protocol for each cell line of iPSCs. In the future, the donor site may be selected on the basis of its reprogramming potential and differentiation tendency to meet the requirements for different diseases and to fit in various microenvironments.

### 2.4. Aging of the Donor Cell and Cell Senescence

Epigenetic changes also contribute to disease development. The pathogenesis of AMD involves environmental and genetic factors. AMD has two types: the wet form and dry form; the former is categorized with choroidal neovascularization, and the other type is known to show geographic atrophy [44]. Current studies have shown that AMD is related to epigenetic changes that connect environmental factors to disease development [45]. Histone methylation, phosphorylation, acetylation, and other epigenetic changes caused by aging, cigarette smoking, oxidative stress, and UV light ultimately lead to abnormal gene expression [46,47,48]. Hypomethylation of interleukin-17, methylation of antioxidant glutathione S-transferase isoforms mu1 and mu2, and other epigenetic molecular modifications are related to AMD [49,50].

Epigenetics also contributes to cellular senescence. Current studies have indicated that epigenetic changes including altered patterns of histone posttranslational modifications and DNA methylation, replacement of canonical histones with histone variants, and altered noncoding RNA expression are important factors for aging [51,52,53]. Aging also has a considerable impact on stem cell therapy. iPSCs from elderly mice showed lower reprogramming efficiency and proliferative ability than younger mice. Dermal fibroblasts from 1-year-old mice have a fivefold lower frequency of colonies with alkaline phosphatase expression, a stem cell marker, than those from juvenile mice, indicating a significant age-related decline in reprogramming efficiency [43,54,55]. Namely, senile patients will receive relatively imperfect iPSCs reprogrammed from their own aged somatic cells. This phenomenon is more conspicuous because AMD is more prevalent in the elderly [56]. The way in which to compensate and preclude the age-related decline in potency of iPSCs deserves further research.

Epigenetic modifications play an important role in cell differentiation, disease development, and cellular senescence and have been utilized as a “controller” in reprogramming or an entry point for disease treatment. Kim et al. rescued the poor blood-forming activity of neural progenitor cell-derived iPSCs by treating them with trichostatin A and 5-azacyzidine, an inhibitor of histone deacetylase and a methylation-resistant cytosine analogue, respectively [43], revealing the considerable potential of epigenetic modifications in regenerative medicine. One of the popular issues in these years has been remodeling of epigenetics profile during cell reprogramming. Introducing a specific six-factor gene cocktail to an adult somatic cell can reprogram it into an iPSC with reversion of the gene expression profile related to cell senescence [40,57]. This finding has been applied to animal models. Mice with a G609G mutation in the gene *Lmna*, which leads to progerin accumulation, were short-lived and displayed accelerated multiple organ aging [58,59]. Ocampo et al. utilized cyclic in vivo introduction of OSKM in G609H mutant mice and successfully extended the lifespan of their animal subjects. In addition, electrocardiography showed partial resolution of bradycardia. Aging changes in the histological findings in skin, spleen, kidney, and stomach were improved with cyclic induction of OSKM [60]. This remarkable research informed us of a hypothesis that partial reprogramming in vivo may slow the aging process and extend organ lifespan. Patients with AMD or other degenerative retinal diseases may be beneficiaries of either reversion and erasure of aging epigenetic markers during reprogramming or improved iPSC efficiency and proliferation potential led by epigenetic modifications.

### 2.5. Limitations and Current Difficulties

#### 2.5.1. Irreversible Senescence Change and Genetic Defect

Although epigenetic changes partially contribute to cellular aging, the mechanism of aging involves more factors including DNA damage, increased reactive oxygen species (ROS) production, telomere shortening, and defects in the nuclear envelop [61,62,63,64]. Some aging hallmarks and genetic defect may not be repaired or erased and can even lead to failure of transplantation. Rapid shortening of telomeres and increased p21 expression, which caused cell growth arrest, were noted by Kokkinaki et al. Due to the limitation of unexpected rapid senescence, only early passages can be used for regeneration [24]. As another example, an embryonic fibroblast from a p53-deficient mouse may acquiesce in a reprogramming process without p53-dependent apoptosis and become an iPSC with persistent DNA damage, resulting in chromosomal instability and potential tumorigenicity [65]. Genomic analysis can be performed before transplantation, but the relationships among copy number alteration, epigenetic variation, and tumorigenicity are still under investigation.

#### 2.5.2. Long-Term Survival of Grafted Cells

Long-term survival of grafted cells is a goal of transplantation; however, eyes with degenerative disease have a microenvironment associated with oxidative stress and leukocyte infiltration, which is unfavorable for grafted tissue. A follow-up report showed a survival time of iPSC-retina in its hosts of at least 5 months (rat) to over 2 years (monkey) [66]. A 4-year follow-up report of a patient with grafted iPSC-derived retina indicated that the graft had survived, and it was able to support photosensors [67]. Effort has been devoted to promoting the survival of grafted cells. Rho-associated protein kinase (ROCK) inhibitor was found to promote iPSC retinal cell survival and cell adhesion in vitro, and no toxic effect was noted in a monkey model [68]. We need longer observation periods to evaluate the lifespan of grafted cells in general as we expect that iPSC therapy can slow disease progression and improve vision; however, a rescue algorithm might need to be established to deal with graft tissue failure.

Provided with their advantages including the capability to differentiate into targeted somatic cells, provision of a stable and indisputable cell source, relief from immunosuppression and related complications, potential for disease model study, and promotion of personalized drug development, further achievements of iPSCs in different aspects can be expected.

## 3. Mesenchymal Stem Cell (MSC)

### 3.1. Current Application and Development

MSCs, first discovered by Friedenstein et al., can divide and produce progeny through specific and dedicated pathways and ultimately become end-stage cells characterized by unique tissue types. According to the International Society of Cellular Therapy, MSCs should meet the following criteria: (1) MSCs must be plastic-adherent when cultured in standard and appropriate conditions. (2) At least 95% of MSCs must express CD105, CD73, and CD90, which are glycoproteins MSC markers. Negative expression of CD45, CD34, CD14 or CD11b, CD79a or CD19, and HLA class II is also required. (3) MSCs must be able to differentiate into stem cell lineages such as osteoblasts or adipocytes, in vitro [69,70,71]. In different parts of human tissue, we found MSCs, including bone, cartilage, tendon, adipose, muscle, and neural cells [72,73]. In all MSC sources, adipose stromal cells (ASCs) have aroused interest because fat tissue is considered medical “waste” and is easy to harvest. One gram of adipose tissue contains 500,000 to 1 million cells, providing a stable and near-inexhaustible supply [20].

**Figure 2:**
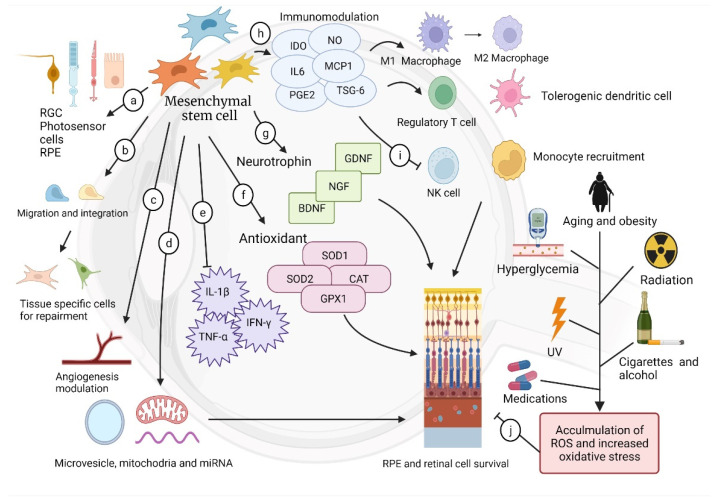
(a) MSCs can differentiate into retinal cells as MSC-derived organoids for replacement therapy. (b) After being delivered into eyes, MSCs are shown to be able to migrate and integrate into the inflammation site, then differentiate into tissue-specific cells to repair injured tissue. (c) MSCs exert angiogenesis or anti-angiogenesis, depending on the microenvironment. (d) Interaction between MSCs and other cells relied on release of microvesicles, mRNA, or mitochondria as cellular communication signals. (e) MSCs are known to reduce inflammation factors. (f) MSCs can upregulate gene encoding for antioxidant enzymes to eliminate ROS. (g) Neurotrophins support neural survival and growth. (h) MSCs cytokines modulate immune response and accelerate generation of M2 macrophage, tolerogenic dendritic cells, and regulatory T cells. (i) MSCs inhibit natural killer cell proliferation and monocyte recruitment. (j) ROS from the environment, personal behavior, and lifestyle accumulate as aging and lead to oxidative stress of the retinal tissue.

MSC-derived retinal cells have been studied. Photosensor-like cells that express rhodopsin and CRX genes can be differentiated from trabecular mash mesenchymal cells (TMSCs) [74], and human adult dental pulp stem cells can express the cell marker rhodopsin after being cultured in conditioned media [75]. Vossmerbaeumer et al. induced expression of RPE markers (estrophin, cytokeratins 8 and 18, and RPE 65) in ASCs [76]. MSC-derived RPE has also been used to improve visual acuity in mouse models. Li et al. utilized human amniotic epithelial stem cells as seed cells and a source of RPE cells for replacement [77]. However, the application of MSC-derived organoids in human models still requires further evidence. Although MSC-derived organoids are not generally recognized in clinical use, MSCs possess diverse mechanisms and repair damaged tissue in amazing ways. MSCs are able to promote cellular survival; modulate angiogenesis; and reduce inflammation through release of cytokines, exosomes, or other neurotransmitters. Moreover, MSCs are known to migrate, integrate, and differentiate into local components of injured sites for tissue repairment [78,79] (Figure 2).

### 3.2. Homing and Migration

To date, most studies utilizing MSCs to treat retinal disease employ a cellular suspension or MSC-conditioned medium and deliver MSCs by either subretinal or intravitreal injection. Subretinal injection has the advantage of facilitating direct contact to the retina, allowing easy migration, while intravitreal injection can deliver a larger number of cells and has a decreased risk of retinal trauma [80]. Regardless of the delivery mechanism, an intriguing issue for clinical application of MSCs is migration capability. Sarbash et al. systemically delivered BM-MSCs labeled with 99mTc exametazime to rats with coronary artery occlusion and traced their distribution. One week later, histological examination showed aggregation of labeled cells in the infarction zone and nearby, indicating that BM-MSCs tended to be attracted and recruited to the ischemic area [81]. Synthesis of matrix metalloproteinase 2 (MMP-2), membrane type 1 MMP (MT1-MMP), tissue inhibitor of metalloproteinase 1 (TIMP-1), and TIMP-2 was noted in BM-MSCs. Silencing of MMP-2 and MT1-MMP, which contributes to hMSC-mediated collagenolysis, can impede BM-MSC migration, while shutdown of TIMP-1 strengthens it. Inflammatory cytokines, including TGF-β1, IL-1β, and TNF-α, facilitated BM-MSC migration and extravasation into injured tissue by upregulating MMP-2 and MT1-MMP [82,83]. Barzelay et al. found upregulation of CXCR4 in ASCs and SDF-1 in the RPE, both promoting cell migration, with exposure to oxidative stress; in contrast, ASCs free from oxidative stress showed a significantly decreased migration capacity [84]. In addition to inflammatory cytokines and oxidative stress, stimulating factors in MSC migration involved eclectic dimensions from growth factors, mechanical stretching to adhesion molecular [85,86,87]. After migration, MSCs can either release supporting and neurotrophic cytokines or differentiate into tissue-specific cells to repair the wound site [78]. Huo et al. delivered MSCs to the subretinal space of NaIO3-induced retinal degeneration of rat eyes. Two weeks after the transplantation, they found grafted MSCs survive and express cell markers of RPE and photosensor cells [88]. A similar conclusion was obtained by Castanheira et al. as they transplanted MSCs intravitreally into laser-damaged rat eyes. The outcome showed survival of grafted cells for 8 weeks with expression of rhodopsin and parvalbumin, a cell marker of bipolar and amacrine cells, indicating the migration, integration, and differentiation of MSCs [89]. The inner limit membrane (ILM) has been considered a barrier that obstructs migration and incorporation of intravitreally transplanted cells into the neural retina, but disrupting neither the basement membrane nor the glial endfeet enhanced grafted cell migration [90]. Several strategies were published to enhance MSCs migration including CXCR4 modification [91], exposure to hypoxia during culture [92], Lentiviral overexpression of Nur77 or Nurr1 [93], etc., but no satisfying application in retinal disease to date. It deserves more effort to combine respective advantages of current delivery routes and find out an ideal way carries adequate grafted cells and less invasiveness.

### 3.3. Paracrine Effect

A paracrine effect with cytoprotective properties of mesenchymal cells was first described by Gnecchi et al., who found that paracrine mediators released by the MSCs can prevent ventricular remodeling and reserve cardiac function [94,95]. MSCs stimulate and modify cell behavior through secretomes, consisting of microvesicles, exosomes, proteins, and cytokines, to partially repair injured tissue without direct attachment of itself to the lesion site [96]. The interaction and signaling passage between one cell and another are the so-called paracrine effect. Such an epochal finding makes MSC therapy a rapidly advancing subfield of regeneration therapy. Intramuscular injection of human umbilical cord MSCs (hUCMSCs) in rats with dilated cardiomyopathy improved the left ventricular ejection fraction compared with rats in the control group, suggesting that a paracrine effect likely promotes myocardiocyte survival and is therapeutic [97]. With regard to retinal disease, a paracrine effect has been shown to promote survival of injured RGCs and to reduce nerve gliosis, whereas a paracrine factor could provide neuroprotection and neurotrophy. More fascinating features are discussed below [98,99,100].

### 3.4. Immunomodulation

Immunomodulation has been noted because solute factor released by MSCs placed suppression on maturation and differentiation of CD4+ and CD8+ T cells, B cells, and nature killer cells, suggesting anti-inflammatory abilities in retinal disease [101,102,103]. When situated in an inflammatory microenvironment, MSCs can modulate the immune response by releasing mediators including IDO, IL-6, and PGE2 [104]. Perforin-dependent apoptosis reactions of MSCs are induced by cytotoxic cells, and apoptotic MSCs suppress the immune response by secreting indoleamine 2,3-dioxygenase after being engulfed by host macrophages [105,106]. Furthermore, MSCs increase the generation of M2 macrophage and regulatory T cells, promote tolerogenic dendritic cells, and suppress natural killer cell proliferation [107,108,109]. Downregulation of monocyte chemotactic protein (MCP)-1, a chemokine recruiting monocytes toward the inflammation site, was noted after exosome treatment [110,111,112]. In addition, MSCs have been shown to be able to migrate to the damaged site to regulate inflammation and repair the tissue [113]. Proinflammatory cytokines lead to breach of the blood–retinal barrier and cause fluid accumulation in the retina, which are major causes of RD and AMD [114,115], turning out to be an ideal target for treatment. Hermankova et al. administered the proinflammatory cytokines IL-1β, TNF-α, and IFN-γ intravitreally to rats. When MSCs were introduced intravitreally, expression of genes for the abovementioned proinflammatory cytokines and the subsequent macrophage infiltration were reduced, highlighting the immunomodulatory characteristics of MSCs [116].

### 3.5. Angiogenesis Modification

For a long time, vitreoretinal interfacial neovascularization has been a difficult problem and the most common cause of moderate to severe vision impairment as it may lead to tractional retinal detachment and vitreous hemorrhage. It can be categorized into two types: retinal vascular disease and subretinal neovascularization. The former is characterized by leakage or neovascularization of retinal vessels and includes DR, retinal vein occlusions, and retinopathy of prematurity (ROP), while the latter is associated with abnormal vascular growth in the avascular subretinal space, which is mainly noted in pathologic myopia or AMD [117,118]. MSCs are known to participate in angiogenesis—cardiologists first found that MSCs release VEGF in rats with myocardial ischemia; furthermore, intravenous injection of MSCs was shown to improve cardiac function through angiogenesis [119,120]. Finding of angiogenetic characteristics of MSCs seems to be in conflict with the aims of treatment of retinal disease, since neovascularization in the retina is not preferred. Nevertheless, MSCs have been shown to exert either anti-angiogenic or angiogenetic effects, namely, they modulate angiogenesis depending on the microenvironment in which they are transplanted. Kasper et al. found that mechanically stimulated MSCs promote angiogenesis through the VEGFR signaling cascade and fibroblast growth factor receptor pathway [121]. Their anti-angiogenic properties were noted because intravitreal administration of MSCs to mice with DR led to elevated intraocular level of Thrombospondin-1 (TSP-1), an anti-angiogenic factor [122]. Kim et al. demonstrated that systemically injected amniotic membrane-derived MSCs could produce transforming growth factor-β (TGF-β1), directly inhibiting endothelial cellular proliferation in an oxygen-induced retinopathy model in vivo [123]. Gaining further insight regarding the interaction between the microenvironment and the anti- or pro-angiogenic properties of MSCs could be a rational approach to provide MSC therapy with improved stability and predictability.

### 3.6. Neural Protection and Growth Supplement

The paracrine effect of MSCs is also featured with neural protection, injury amelioration, and growth supplement. Arnhold et al. transplanted MSCs through subretinal injection into rhodopsin knockout mice, and the outcome showed that the photoreceptor nuclei density in MSC transplanted mice was significantly higher than that in mice in the control group [124]. MSCs, MSC-derived exosome (MSC-Exos), and microvesicles have been proven to alleviate retinal laser injury. By releasing neurotrophins, such as nerve growth factor and glial cell line-derived neurotrophic factor, MSCs can extend neural cell survival and promote neural repairment. Expression of neurotrophic factor genes in vivo by grafted MSCs could be confirmed by quantitative RT-PCR [125,126].

### 3.7. Antioxidation

Oxidative stress originates from accumulation of ROS beyond the capacity of antioxidant defenses to deal with them. ROS include oxygen radicals such as peroxides, superoxide, hydroxyl radicals, and ozone, which is prone to become radical [127,128]. ROS is originated from bioenergetics to metabolism, including UV light, ionized radiation exposure, smoking or alcohol consumption, obesity, and certain medications, playing important roles in tissue damage, inflammation, carcinogenesis, and retinal disease [129,130,131,132,133,134,135]. Despite the notorious negative effects of ROS on the human microenvironment, MSCs have been confirmed to demonstrate anti-oxidative properties. Ohkouchi et al. co-cultured A549 cells with MSCs, and hydrogen peroxidate was added as oxidative stress. The outcome showed that MSCs promoted the survival of A549 cells through STC1 (Stanniocalcin 1) upregulation, while A549 cells co-cultured with anti-STC1 antibodies blocking STC1 showed no resistance to oxidative stress [136]. AASCs have been used as a treatment for oxidative injury in mouse models. They were injected into the subretinal space of mice receiving systemic NaIO3, and the outcome showed improved preservation of the outer nuclear layer and photosensor cells compared with mice that received phosphate-buffered saline [84]. Previous studies have partially illustrated the mechanism of antioxidate. MSCs isolated from bone marrow (BMSCs) express the genes SOD1, SOD2, CAT, and GPX1, which code for enzymes that eliminate ROS [137]. Heat-shock protein 70 (HSP70) expression also contributes to resistance to oxidative injury [138]. In summary, MSCs promote neural survival as it is able to secret neurotrophin, exert antioxidants, and reduce inflammation.

### 3.8. Mitochondria Donation, Cellular Signal, and Cell-Free Therapy

Mitochondria generate chemical energy for biochemical reactions. Most mitochondrial proteins are encoded and imported from nucleus DNA, maintaining mitochondrial functions. Some reports show these nuclear-encoded proteins play crucial roles in mitochondria-mediated repair for ischemic injury through reducing oxygen consumption [139], inducing synthesis of aldehyde dehydrogenase [140], and increasing the activity of cytochrome c oxidase [141], etc. Mitochondria contains its own DNA, known as mitochondrial DNA (mtDNA), which encodes 13 proteins, while nuclear genes encode most of the mitochondrial proteins [142,143]. Dysfunction of mitochondria is a sign of cellular senescence, and mitochondrial injury is a causative factor of RPE cell death and degenerative retinal disease. Both aging and hyperglycemia lead to oxidative stress, damaging mitochondria and accelerating AMD and RD development. Increased hydrostatic pressure, a major cause of glaucoma, also leads to mitochondrial function disturbances and RGC loss [139,140].

A creative way that MSCs assist injured neural cells has encouraged widespread discussion. MSCs can deliver their own mitochondria to damaged cells through different approaches to promote their regeneration and repair. This phenomenon was first described by Spees et al., who cocultured mitochondrial gene-depleted cells with MSCs. The mutant cells with enhanced mitochondria showed elevated ATP levels and significantly decreased lactate production, a byproduct of anerobic respiration, and expression of mitochondrial proteins [141]. Past studies have indicated several ways that mitochondria can be delivered including via tunneling nanotubes (TNTs), gap junctions, or exosomes [142,143,144]. This donation is observed in ocular cells such as the corneal endothelium, RPE, and photoreceptors [145]. Furthermore, MSCs are able to release survival signals to promote mitochondrial biogenesis in retinal cells. Kim et al. overexpressed PEDF, an antioxidative factor, in placenta-derived MSCs through a nonviral gene delivery system and cocultured it with an oxidative stress-damaged RPE. The outcome showed enhancement of biogenesis regulators including NRF1, PPARGC1A, and TFAM, which are required for respiration, mitochondrial transcription, and biogenesis [146]. Such a unique rescue mechanism expounds the potential advantages for future stem cell medicine.

The therapeutic functions mentioned above including antioxidative properties, immune and angiogenesis modulation, neural protection, and growth supplementation are based on signal and paracrine from MSCs, instead of delivering MSCs to the lesion site. A new concept of stem cell therapy was also invented. Cell-free therapy using MSC-derived products or conditioned media to rescue injured cells has been applied to retinal disease; this therapy omits the complicated process of cell migration and integration. Mead et al. have demonstrated that exosomes of BM-STC-containing miRNA can promote RGC survival [147]. Furthermore, secretomes from trabecular meshwork (TM) stem cells have been shown to stimulate TM regeneration and enhance RGC survival by upregulating COX2-PGE2 [148]. hUCMSC-derived exosomes can also attenuate subretinal fibrosis, and ASC-derived exosomes can slow RD progression [149,150].

Cell-free therapy simplifies the storage procedure and has less concerns about loss of product activity. It avoids complications such as emboli formation, tumorigenicity, and infectious material transmission during introduction of live cells into the human body. Time-consuming and expensive procedures including MSC isolation and expansion are no longer needed, making this treatment available in acute disease and improving propagation. The more features of these signaling pathways are discovered, the more promising therapies can be expected, and cell-free therapy will hold a special place in regeneration therapy.

### 3.9. Genetically Engineered MSCs

With the aim of equipping MSCs with specific functions preferred for treatment or promotion of differentiation and survival, genetically modified MSCs have been evaluated. Genetic modifications can be generally made with viral or non-viral vectors [151]. Knowing that erythropoietin (EPO) is a key factor in retinal cell differentiation and rescue of dying photosensor cells, Ding et al. have reported that MSCs induced with an EPO gene fragment showed improved potential to differentiate into photosensor cells [152]. BM-MSCs modified with brain-derived neurotrophic factor (BDNF) overexpression were intravitreally delivered into Rd6 mice. MSC-BDNF was detected in the deep retinal layer, while normal BM-MSCs were restrained in the vitreoretinal surface, indicating that their migration may be promoted by gene modification. Furthermore, BDNF secretion persisted for 4 weeks, and BDNF did achieve neuroprotection by stimulating the Akt and MAPK-related pathways [153]. Other studies that focus on the application of genetically modified MSCs in retinal disease are still in process and may provide further breakthroughs in the coming years.

Despite the abovementioned advantages of MSC therapy, including inflammation and angiogenesis modulation, antioxidation, paracrine and neurotrophic effects, and cell migration, clinical applications still face several challenges and limitations.

### 3.10. Limitation and Challenges

#### 3.10.1. Persistent Survival of MSCs

Persistent survival of the grafted cells is a crucial factor for long-term disease control and a goal we are striving toward. Death of most grafted cells occurs in the first week after transplantation. The head-on stress for MSCs is detachment of the culture plate and preparation as a cell suspension. Loss of matrix support may lead to anoikis, which is apoptosis induced by inadequate or inappropriate cell–matrix interactions [154]. Then, grafted cells are placed into an inflammatory environment where nutrition and oxygen are deprived. Leukocytes and macrophages are recruited and produce ROS, causing direct damage to the grafted cells [155,156]. Several strategies have been studied to promote survival. Past studies have indicated that pretreatment with atorvastatin, melatonin, and microRNA-125b can benefit grafted cell survival [157,158,159]. Genetic engineering can be employed in this aspect too. Toll-like receptor 4 (TLR4) is a G-coupled protein that triggers the pathway of apoptosis during ischemic injury, and TLR4-knockout MSCs have shown improved survival and resistance to ischemia [160]. Upregulation of hypoxia-inducible transcription factor-1α, which silences PHD2 expression, can promote BM-MSC survival [161]. With increasing methods to enhance graft survival, more comparisons of the efficiency, safety, accessibility of the ameliorating technique, and associated expenses between different survival-improving methods are needed to make MSC therapy more prevalent.

#### 3.10.2. Replicative Senescence and Age-Related Impacts

As MSCs replicate, decreased differentiation potential, known as “replicative senescence”, has been noted. Epigenetic changes may contribute to this phenomenon, while karyotypes remain unaltered during replication. Replicative senescence occurs, even within first passage in some colonies [162]. As a result, donor age, namely, patient age in autologous transplantation, is important. MSCs isolated from elderly donors (>60 years) are characterized with reduced viability and differentiation potential compared with those from younger donors (<30 years) [163]. Human platelet lysate (HPL), which is derived from human platelets, is a growth supplement media for MSCs cultures. Proliferation of MSCs with HPLs from younger donors was significantly higher than MSCs cultured with HPLs from older donors [164], indicating the influence of aging is not only observed in MSCs but also in the culture environment and is a major uncertainty in cell therapy. It is technically possible to derive HPL from only younger adults, but this involves legal and ethical issues; however, allogenic transplantation of grafts from a young donor to an older patient must meet the challenge of immunosuppression-related complications. Methods of overcoming the senescence of MSCs from an elderly donor are worth further study.

#### 3.10.3. Other Challenges

In addition to these predicaments, there is no standard measurement to evaluate the quality of MSCs and no consensus on clinical protocols. Even groups of MSCs from the same organ are composed of a heterogenous population, which increases the uncertainty of their differentiation potential or cellular behavior [165]. There are no standard protocols regarding how many grafted cells should be transplanted and how to deliver the MSCs or their derivative products. Although it is widely recognized that subretinal delivery can provide a more precise and efficient route for administration of ocular drugs, gene therapy, and cell therapy than intravitreal injection, whether delivery routes should be modified depending on the lesion site or disease properties requires more extensive and rigorous testing [166,167].

## 4. Conclusions

In the past decades, regenerative therapy was restricted by its rarity and ethical concern regarding embryonic or fetal stem cells. However, iPSC and mesenchymal cell therapy started a new chapter. Stem cells are no longer a scarce resource because they can be isolated from non-vital organs from patients themselves. iPSCs can differentiate into retinal cells and be transplanted for vision improvement. They can also serve to build disease models, develop individualized therapies, and promote drug selection. The latest findings have uncovered the reversion of aging epigenetic markers during reprogramming and may inspire a new way to cure degenerative disease. MSCs exist in a variety of tissues in our body, with subtle variations between different tissues. MSCs can be induced into somatic cells for replacement therapy, but their paracrine function is more surprising, promoting cell growth, dealing with oxidative stress, and modulating angiogenesis. MSCs also pass signals between different cells by exosomes or even mitochondrial donation. Cell-free therapy utilizing MSC-related products or conditioned media have recently emerged and have the advantages of being easily purified and stored. Nonetheless, stem cell therapy in retinal disease has met several challenges. Grafted cells derived from iPSCs or grafted MSCs must survive in a fluctuating environment altered by disease progression. Current studies have shown survival of grafted tissue with RPE function, but the improvement in visual acuity was far from “clinically meaningful”. Every single improvement in stem cell research, including cell isolation, storage, culture media, transplantation technique, post-transplantation care, and genetic modification, can contribute to further improvements in therapy. With more novel and intensive studies, we can look forward improving the future of suffering patients.

## Data Availability

Not applicable.
